# Phage Therapy in Livestock and Companion Animals

**DOI:** 10.3390/antibiotics10050559

**Published:** 2021-05-11

**Authors:** Celia Ferriol-González, Pilar Domingo-Calap

**Affiliations:** 1 Department of Genetics, Universitat de València, 46100 Valencia, Spain; cefegon@alumni.uv.es; 2 Institute for Integrative Systems Biology, I2SysBio, Universitat de València-CSIC, 46980 Valencia, Spain

**Keywords:** phage therapy, veterinary medicine, antimicrobial resistance, antibiotics

## Abstract

The irrational use of antibiotics has led to a high emergence of multi-drug resistant (MDR) bacteria. The traditional overuse of antibiotics in the animal feed industry plays a crucial role in the emergence of these pathogens that pose both economic and health problems. In addition, antibiotics have also recently experienced an increase to treat companion animal infections, promoting the emergence of MDR bacteria in pets, which can reach humans. Phages have been proposed as an alternative for antibiotics for the treatment of livestock and companion animal infections due to their multiple advantages as adaptative drugs, such as their ability to evolve, to multiply at the site of infections, and their high specificity. Moreover, phage-derived enzymes may also be an interesting approach. However, the lack of regulation for this type of pharmaceutical hinders its potential commercialization. In this review, we summarize the main recent studies on phage therapy in livestock and companion animals, providing an insight into current advances in this area and the future of treatments for bacterial infections.

## 1. Introduction

The discovery of antibiotics was a revolution in medicine, as they have saved countless lives. However, their indiscriminate use has led to the emergence of multi-drug resistant (MDR) bacteria. This problem is now a major global threat and poses a serious challenge in the search for alternative therapies against MDR bacteria [1]. A key factor determining the high rate of MDR spread is the irrational use of antibiotics [2]. The World Health Organization describes the rational application of a drug as when patients receive an appropriate drug for the appropriate indications in the doses personally and individually required for an indicated period of time, implying the lowest cost and with suitable information available, among other measures [3]. The problem of antibiotic resistance causes serious health and economic losses to society worldwide [4]. For example, in the European Union, 25,000 patients die each year due to infections caused by MDR bacteria, with an associated economic cost to society of about 1.5 billion annually [5]. In addition, the COVID-19 pandemic has led to an increase in antibiotic prescription, exacerbating the prospects for antimicrobial resistance [6,7,8].

The agro-food industry plays a crucial role in the emergence of MDR bacteria. In developed countries, livestock farming accounts for about 50–80% of total antibiotic use [9,10]. Moreover, the highest rates of antibiotic resistance are detected in tetracycline, sulfonamide, and penicillin, antibiotics commonly used in the animal feed industry [11,12]. Different factors seem to play a role in the prevalence of MDR microorganisms. Farm size has been associated with higher rates of antimicrobial resistance, which may be related to differences in farm management (hygiene and feeding practices). Antibiotic usage in farms contaminates water with MDR microorganisms that even reach wild species, where the existence of these microorganisms has also been evidenced [12]. Antimicrobials in animal production play an important role in MDR bacteria in humans. Most of the antimicrobials used in food-producing animals are closely related or identical to human antimicrobials. Extensive usage of antibiotics in the food industry can increase the spread of MDR bacteria that can reach humans through food or other routes [13]. Antibiotics in livestock are not exclusively treatments for bacterial infections but have also been widely used as growth-promoting factors since 1951 [9,14]. Indiscriminate use of antibiotics has been linked to higher levels of antibiotic-resistance genes that are also transferred to bacteria infecting humans. Denmark banned Avopracine in 1995, one of the antibiotics used as a growth promoter in livestock, followed by the EU five years later, banning 11 antibiotics for growth promotion. Therefore, nowadays antibiotics usage in the EU varies greatly among EU members, with Italy and Spain leading the way [9].

Despite the fact that companion animals are not linked to the human food chain, they can also act as reservoirs of MDR bacteria [15], and their proximity to humans makes them a potential focus for cross-transmission of zoonotic bacteria, including MDR strains that pose a potential threat to human health [11,16]. Nowadays, the range of species that can be considered as companion animals has increased significantly, including a wide diversity of species (rodents, guinea pigs, reptiles, etc.). This increases the likelihood of human contact with microorganisms and thus the possibility of transfer of dangerous pathogens [16]. Social concern for health care has grown in recent decades, especially in urban areas. Due to their easy availability, the most commonly used antimicrobials in companion animals are human antibiotics, which increases the emergence of MDR strains [6,17], and they become potential reservoirs. Due to the increase in MDR strains that can affect humans, which is caused by misuse of human antimicrobials in animals [11], it has become necessary to investigate new alternatives to animal-only antibiotics. Here, we propose bacteriophages (phages), viruses that infect bacteria, as a promising tool against MDR bacteria and as an alternative treatment in the fight against pathogenic bacteria.

## 2. Phages as a Promising Alternative Therapy against MDR Bacteria

Phage diversity is enormous, being the most abundant entity in the biosphere [18]. They can be found in a wide range of environments, including extreme ones [19]. Despite the variation found in phages, they present two main biological cycles, with some modifications: lysogenic cycles (temperate phages), in which phage DNA integrates into the host genome as a prophage; and lytic cycles (lytic or virulent phages), in which phage multiplies inside the host bacteria and releases new phage particles by lysing the host cell [20,21]. Lytic phages are especially interesting for phage therapy due to two main reasons: firstly, because their cycle leads to bacterial death [22] and secondly, because they lack integrases and other related enzymes, thus avoiding horizontal gene transfer [23].

### 2.1. Phage Therapy Overview

Phages were discovered independently by the English military physician Fredrick W. Twort in 1915, and the Canadian microbiologist Félix H. d’Hérelle in 1917 [22,24]. Phages were successfully tested by d’Hérelle early on to treat dysentery and cholera. However, World War II and the discovery of antibiotics led to the abandonment of phages as a therapeutic tool in Western countries [25]. Nowadays, due to the emergence of MDR bacteria, phage therapy has re-emerged and is considered a potential therapeutic alternative to antibiotics [21,26].

**Figure 1:**
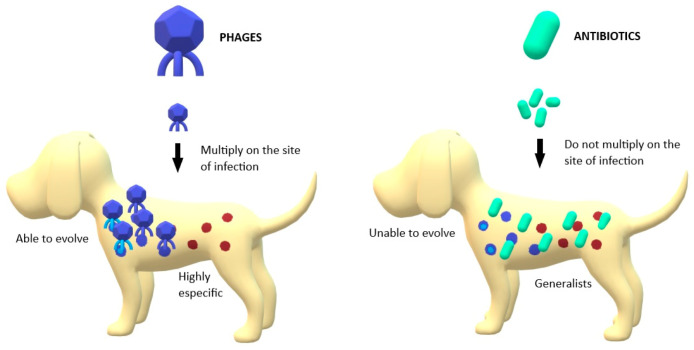
Main differences between phages’ and antibiotics’ action against pathogenic bacteria; Phages are able to multiply in the site of infection, unlike antibiotics. Phages are able to evolve with bacteria while antibiotics are static structures. Phages are highly specific in their targets while antibiotics are generalists.

Phage therapy presents some potential advantages for treating bacterial infections. They are able to kill bacteria regardless of whether the bacteria are resistant or not [27]. In fact, MDR *Acinetobacter baumannii* strains have been shown to be more susceptible to phage therapy than other non-MDR strains [26,28]. Furthermore, phages multiply at the site of infection (Figure 1), increasing in number during the infection process and making it likely that a single (or few) doses will be sufficient to have the desired effect compared to antibiotic treatment. This makes hard-to-reach infections more accessible to phages than to antibiotics if sufficient numbers of phages are applied initially [29]. Phages are also highly specific (Figure 1), implying that they may not affect the commensal bacteria of the host [30]. If a broader spectrum is required to treat an infection, it is possible to use combinations of phages, known as phage cocktails [31]. Phage cocktails are a growing strategy in phage therapy to improve the results of monophage therapy. In addition to extending the utility of phage formulations, phage cocktails may also be useful in preventing the development of phage-resistant strains during individual treatments [32]. Another advantage of phage therapy is that phages can be considered as "adaptive drugs" (Figure 1). Bacteria and phages have co-evolved in nature for millions of years. This may also occur in phage-treated infections, where this coevolution makes the treatment adaptive [26,33]. Furthermore, phage cocktails tend to decrease the emergence of bacterial resistance compared with the application of individual phages [21], as has been shown in other combination therapies [34,35].

An important remark in phage therapy is that the pharmacokinetics of phages is more complex than that of traditional antibiotics, and there may be differences between phages in terms of their persistence and ability to replicate. They may also interact differently with the immune system, and their efficacy would also depend on the characteristics of the infection, such as its location, abundance, and bacterial composition. Their interaction with many plasma proteins is also largely unknown. Thus, pharmacodynamics and pharmacokinetics considerations become more complex with phages or phage-derived combinations [36] and should be especially important in defining the administration process and dosing.

Regarding phage delivery strategies, oral administration has limitations due to the poor stability of phages in acidic environments such as the stomach. Many authors have described this problem, proposing as a solution the administration of phages with buffering compounds. This would significantly increase phage survival [16]. Other strategies such as nano- and microencapsulation allow controlled or sustained release of phages at the site of infection or increase the time of phage circulation [37]. Another alternative suggested by some authors is rectal application. It has been successfully tested in rabbits using a nonionic surfactant to increase the presence of phages in the blood [38]. In another study, the authors tested a form of phage suppository. This method ensured the presence of phage particles in the foci of infection directly in contact with the preparation [39]. For the treatment of pulmonary infections, phage inhalation has been proposed as an alternative and seems particularly promising, but more research on this method of application is still needed [40]. To treat skin and wound infections, the application of phage therapy directly on the skin has been successfully tested. One example is the treatment of *Klebsiella pneumoniae*, a pathogen predominantly associated with burn-wound infections. In one study, both a phage therapy and a phage cocktail have been tested in a murine model. Both groups showed a significant reduction in bacterial load compared to the untreated group of mice. The growth that received the phage cocktail showed the maximum reduction (*p* < 0.01) [41].

### 2.2. Phage-Derived Enzymes

Another phage-related strategy already tested in animal models that has been proposed as an alternative to antibiotics is the use of phage-derived enzymes. Phages produce various types of enzymes capable of targeting specific bacteria [42]. Among other functions, some enzymes help them to penetrate the bacterial host and participate in bacterial lysis [21,43].

One of the most interesting types of phage-derived enzymes for therapy is lysins. Lysins are very specific enzymes that cleave peptidoglycan bonds and have a bactericidal effect on susceptible bacteria. Lysins are classified into two main groups: endolysins, which are involved in bacterial lysis, and virion-associated lysins (VALs), which are involved in entry into the host cell, allowing injection of the phage into it [42]. Endolysins are synthesized in the cytoplasm of infected bacteria to cause lysis. They are classified into canonical and exported endolysins. The canonical endolysins are considered the most interesting as enzybiotics and require other phage enzymes, the holins [43]. Lysins are especially useful in biofilm degradation [44]. They have been successfully tested in animal models against bacterial infections [45]. One example is the lysine LysSS, tested in a mouse model of systemic infection by *A. baumannii*, showing its potential to treat systemic infection [21,46].

Holins are enzymes that also act in the lytic process involved in cell-wall degradation. When the concentration of holins exceeds a threshold, they form pores in the bacterial membrane and allow the action of endolysins. Holins are generalists and combine with other enzymes that broaden the spectrum of the host strain [21,47,48,49].

Depolymerases are phage-derived enzymes capable of degrading the extracellular substances that form the capsule of many bacteria [50]. There are several groups according to the type of bond they break [47]. Depolymerases have a broad host spectrum that contrasts with lysins, and they are also particularly useful in the elimination of biofilms [42,51]. For example, the effect of Dp42 depolymerase in the *K. pneumoniae*-infected mouse model has also been discussed recently. Dp42 increased the survival rate and significantly reduced the bacterial load in the liver, spleen, and lungs of the treated mouse [52].

The creation and modification of phage proteins is also an interesting approach for the treatment of bacterial infections. Thanks to synthetic biology, bacterial spectrum proteins, bacterial resistance to them, and immunogenicity can be improved [21]. In addition, to reduce the emergence of resistance, an alternative solution would be to develop phage-derived enzyme cocktails.

## 3. Phage Therapy in the Veterinary Field

The use of antibiotics in veterinary medicine has been especially irrational during the last few years. Due to the emergence of MDR bacteria, it has become a major problem in this field. Phage therapy has been proposed as one of the most promising alternatives for the treatment of infections in animals to address this problem. Some examples of veterinary use of phage therapy are summarized in Table 1.

### 3.1. Phage Therapy in Livestock and Other Food-Producing Animals

Livestock may be the field where antibiotic use has become most abused, which has resulted in a major threat. Phage therapy has been successfully tested for the treatment of infections affecting especially cattle and swine [69]. In this section, we will mention some recent studies and examples of phage therapy in livestock.

*Staphylococcus aureus* has significant virulence properties, being able to cause several infections in both humans and animals [70]. In addition, *S. aureus* can acquire genes that confer resistance to different antimicrobials, the most frequent strains being methicillin-resistant [71]. This pathogenic bacterium is the cause of mastitis in cattle, a disease that can result in significant economic costs [72]. A recent study proposes the application of three phages against *S. aureus,* using mouse and *Galleria mellonella* models. The results are promising, showing 50% survival of larvae in *G. mellonella* within four days of in vivo treatment with the three phages. In the mouse model, incomplete recovery was obtained after 48 hours post-infection of a single phage [53]. The diminished effect may be explained by the possible interaction of the phages with proteins and lipids present in milk and by the use of a single phage [54]. However, this study demonstrates the potential usefulness of phage therapy as a treatment for mastitis caused by *S. aureus* [53]. Recently, more studies on phage therapy for mastitis in cattle have been developed with positive, significant results using phage cocktails in mouse models. They have shown a decrease in the number of colony-forming units and a significant improvement in mastitis pathology, even greater than in mice treated with a single phage [55,73].

Mastitis is not the only bovine disease that has been proposed to be treated with phage therapy. Metritis is an acute systemic disease that affects cows during the 21 days after calving and has detrimental effects on reproduction [74,75,76]. A cocktail of four *Escherichia coli* phages has been designed and shown to be effective in inhibiting *E. coli* isolates in vitro. Ten phage particles per bacterial cell were sufficient to inhibit the growth of at least 50% of all isolates. These results indicated the potential of the phage cocktail to reduce the presence of *E. coli* in the uteri of dairy cows after calving in order to prevent metritis [56]. However, in vivo results did not show satisfactory results [76,77,78].

Hemorrhagic septicemia is one of the most important epizootic diseases in India, a fatal disease caused by *Pasteurella mutocida* serogroup B:2. The phage PMP-GAD-IND has shown lytic behavior against several strains of *P. mutocida*, including B:2, making this phage potentially useful for phage therapy [57].

Phage therapy has also been proposed to treat swine infections. *Salmonella* sp. can colonize pigs during transport. In a preliminary study, a group of 3- to 4-week-old pigs was administered an anti-*Salmonella* phage cocktail at the time of inoculation with *Salmonella enterica* serovar Typhimurium. Colonization was reduced by more than 99%. In a second experiment, sixteen naïve pigs were introduced into a contaminated pen with four pigs infected with *S. enterica*. One group of them was treated earlier with the phage cocktail. The phage-based treatment showed a significant preventive effect by reducing both cecal and ileal *Salmonella* concentrations [58].

Diarrhea caused by *E. coli* in pigs results in high mortality and morbidity rates as well as decreased growth rate, leading to significant economic losses. Due to the emergence of MDR strains, the search for antibiotic alternatives for this disease is a priority. Phage therapy has been suggested as an alternative due to its important results in human and animal infections [79].

Pigs have been also considered animal models for the design of human treatments. The successful results of these treatments mean that they may be useful for pigs as well. A recent study addresses the use of phages to eliminate bacterial biofilms in chronic wounds in porcine skin explants. Both phage and phage cocktails caused significant reductions in viable cells in porcine skin [59].

It is worth mentioning that cattle are not the only food-producing animal for which phage therapy has been proposed as an alternative to antibiotics. There are several studies developed in poultry. Infections in these animals can cause economic and health problems in our society. *Salmonella* sp., *E. coli*, *Campylobacter* sp., *Listeria* sp., and *Clostridium perfringens* are the main pathogens in the poultry industry. A progressive increase in the number of multidrug-resistant bacteria has been experienced which has encouraged the use of bacteriophages as an alternative treatment for infections caused by these pathogens [80].

Phages have also been proposed as a therapy in aquaculture. One example is *Lactococcus garvieae*, a pathogen affecting several marine fish species. A recent study has characterized three lytic phages against an *L. garviae* strain infecting marine fish species. This opens the possibility of a phage-based treatment against this aquaculture pathogen [81].

Some phage-based products to reduce bacterial contamination of food have already been accepted and commercialized in the USA, Canada, Australia, Israel, and some European countries. Some of these examples are ListShield™, EcoShield™, and SalmoFresh™, developed by Intralyx [82] and PhageGuard S™ products [69,83]. Previous acceptance of these products suggests the potential commercialization of phage-based products for the veterinary field.

### 3.2. Phage Therapy in Companion Animals

Currently, societal concern for companion animal health has increased considerably. This has led to a misuse of antimicrobials to treat infections in companion animals and to an increase in MDR strains [6,17]. However, this societal concern also boosts research into new alternatives for the treatment of infections in companion animals.

A recent study conducted in the United Kingdom provides insight into the views of veterinarians and pet owners on the use of phage therapy in companion animals. Despite the limited sample size (*n* = 20), the study gives an indication of the possible acceptance of phage therapy in these groups. In this study, veterinarians acknowledge their difficulties in finding antibiotics to treat infections and the need for an alternative. The results of the study showed that 75% of the participants would agree with the use of phage therapy for the treatment of infections in companion animals after a brief explanation of what phages are and how phage therapy works. Although veterinarians were more familiar with the concept, pet owners would trust the veterinarians’ advice. This would explain why there were no significant differences between the two groups [84].

Animals traditionally used as models, such as mice, rats, or hamsters, are also considered pets. Therefore, there are many studies on phage therapy using these animals obtained during research on phage-based treatments for humans. For example, a murine model of chronic *Pseudomonas aeruginosa* lung infection was performed to test the efficacy of phage PELP20, demonstrating its potential for phage therapy [60]. Phage therapy has also been tested against MDR *A. baumannii* infections in murine models, showing an increased survival rate of animals when treated with the phage φkm18p [61]. There are also examples of phage therapy for septicemia caused by *S. aureus* in mice, where phage S13’ significantly reduced the severity of infection [62]. Rabbits have also been considered as animal models for testing phage therapy. A cocktail of three virulent phages against *Vibrio cholerae* has been tested in both mice and rabbits to prevent the severe dehydrating disease caused by this pathogen. Oral administration of the phage cocktail against *V. cholerae* reduced pathogen colonization of the intestinal tract, preventing choleric diarrhea [63]. However, these examples all focused on human medicine.

In 2010, the first report of a veterinary clinical trial of a phage-based treatment of infection was published. Ten dogs with chronic *P. aeruginosa* otitis were treated with a cocktail of six phages active against *P. aeruginosa*. The phage preparation was applied directly to the ear canal and massaged for deeper penetration. After 48 h, *P. aeruginosa* counts decreased by 67% (*p* < 0.001), and clinical signs of infection were reduced by 31.1% (*p* < 0.0001) with no adverse events detected. These results suggest the potential of phage therapy in the veterinary setting [64]. A further study focused on phage therapy against *P. aeruginosa*, a major cause of urinary tract and wound infection, isolated and characterized *P. aeruginosa* phages with different lytic activity against 22 *P. aeruginosa* isolates. A phage cocktail composed of *Pbuna*-virus PB1-like phages showed an inhibitory effect on the emergence of phage-resistant variants [65]. 

Phage therapy has been tested to treat other bacterial infections in humans. Extraintestinal pathogenic *E. coli* are bacteria that colonize the human and canine gastrointestinal tract asymptomatically but can cause significant pathology when they infect distal tissues, especially in immunosuppressed patients. MDR in a clonal group of these bacteria, ST131, is increasing worldwide. The HP3 phage was tested in immunosuppressed mice infected with two strains of ST131. Mice treated with the phage showed a significant reduction (*p* = 0.03) of colony-forming units in the liver and kidney. In this case, the phages were administered parenterally [33].

There are more dog infections for which phage therapy has been proposed as a treatment. *Staphylococcus pseudointermedius* causes several diseases in canines, such as urinary tract infections, otitis externa, pyodermal infections, respiratory infections, and reproductive tract infections. Given the high prevalence of methicillin-resistant *S. pseudointermedius* strains, phage therapy is presented as a potentially successful alternative to treat these infections in canines [85] also due to the promising data shown in humans [86]. Several phages have been isolated against *S. pseudointermedius*. In addition, the potential use of staphylococcal endolysins against *S. pseudointermedius* to treat infections caused by this pathogen has also been proposed [85].

Uropathogenic *E. coli* causes urinary tract infections in dogs and cats. An in vitro assay identified five promising phages, four T4-type phages and one with morphological similarity to temperate P2-type phages. This work showed environmental isolation of phages that may be useful in treating canine and feline urinary tract infections [66].

Phage therapy has also been tested for equine diseases. Equine keratitis can be caused by many bacterial species. *P. aeruginosa* is one of them and can cause corneal corruption that can lead to blindness in some cases. Antimicrobial therapy makes its treatment challenging. In one study, a phage cocktail with *Myoviridae* and *Podoviridae* phages demonstrated complete prevention of equine keratitis as well as suppression of this infection, making phage therapy an important candidate for equine keratitis [67].

Reptiles are also part of the wide variety of animals considered as pets. Salmonellosis associated with reptiles as exotic pets has been a growing problem in recent years, caused by poor hygiene after handling reptiles. Because of this potential harm to human health, antibiotic treatment has been tried in reptiles without consistent success and leading to an increase in drug-resistant strains. Phage therapy represents an alternative for the treatment of reptiles. In particular, the phage Felix O1 has been successfully tested in bearded dragons [68].

## 4. Regulation of Phage-Based Products

The recent return of interest in phage therapy increases the importance of legal regulation of phage-based products. Current legislation for pharmaceuticals is defined for industrially manufactured drugs, which can be difficult to relate to phage therapy, considered as a type of evolutionary or personalized medicine due to its unusual pharmacokinetics and evolutionary considerations, posing a major challenge for regulators. The EU is not open to a change in legislation, which is an obstacle to the expansion of new, less conventional therapies [87].

In some Eastern countries, phage therapy practices have never been abandoned, such as in Russia [88] or Georgia [89], and their regulations are being reviewed by some Western countries, making new forms of regulation possible [87]. In Belgium, the first steps are being taken for the regulation of phage therapy by establishing two bases: the elaboration of extended documentation on the phage to be used in the phage product to ensure its quality and the availability of authorized laboratories with phage stock that can ensure that phages raise the quality standards according to technical and scientific knowledge [90]. On the other hand, in France, a specialized committee issued some recommendations for the use of phage-based products according to the Temporary Authorization for Use (ATUn). This is an exceptional procedure allowing the use of a medical product without marketing authorization in the absence of an alternative treatment [91]. In the U.S., owing to the special interest acquired by phages during pandemics for fighting MDR bacteria in hospitals [92], the Food and Drug Administration (FDA) has recently approved phage therapy for COVID-19 patients as a compassionate treatment due to the lack of clinical trials [93]. Regarding their use in animals, phages do not easily fit into existing EU regulations regarding the use of food additives or food processing aids, which is a major obstacle [94]. The EU legislation on veterinary medicinal products and food and feed safety considers different authorizations for substances such as pesticides, biocides, feed additives, or veterinary medicinal products but does not relate to the type of substance. Because of this, phage applications may get different authorizations depending on their application [95].

The lack of specific regulation limits the development of phage therapy, which, at the same time, does not incentivize the interest of the authorities to elaborate these regulations. This increases the importance of research and more clinical trials [87].

## 5. Conclusions

The problem of MDR bacteria has increased disproportionately in recent years due to the misuse of antibiotics. Their use in food-producing animals is particularly abusive despite recent regulations to reduce their impact. The increase of MDR bacteria in this sector causes serious animal health problems and also significant economic losses. In addition, concern for pet care has also meant an increase in the use of antibiotics in companion animals, which implies the emergence of new MDR strains that can cause infections in pets and easily reach humans.

Phage therapy has been proposed as one of the most interesting alternatives due to its properties such as its high specificity, its ability to multiply at the site of infection and to evolve, the potential use of phage-derived enzymes, and also because of its economic cost. Therefore, phage therapy may be an important alternative for the treatment of infections in both livestock and companion animals. Although several studies have been carried out in this area focusing on the treatment of pathogenic bacteria affecting different animals, further efforts are mandatory to enhance the value of phage therapy in animals and will open new avenues for bacterial treatment in the near future. Thus, phage therapy has been explored and proposed as a potential alternative to antibiotics but has not yet been recognized as a therapeutic tool. Interestingly, phage-based products could be easily commercialized, facilitating their use in the market as antimicrobial drugs. However, the potential of phages to control animal infections requires improving the specific regulation of these products, making the need for further research in the field a major concern.

## Figures and Tables

**Table 1:** Some studies about phage therapy in livestock and companion animals.

Animals	Infection	Pathogen	Phages	Paper
Bovines	Mastitis	*S. aureus*	ISP phage	[53]
			Phage cocktail	[54,55]
	Metritis	*E. coli*	Phage cocktail	[56]
	Haemorragic septicemia	*P. mutocida*	PMP-GAD-IND phage	[57]
Swine	Diarrhea	*Salmonella* sp.	Phage cocktail	[58]
	Chronic wounds	Multibacterial biofilms	Phage cocktail	[59]
Mice	Lung infection	*P. aeruginosa*	Phage PELP20	[60]
		*A. baumannii*	φkm18p phage	[61]
	Septicemia	*S. aureus*	Phage s13’	[62]
	Systemic infection	*A. baumannii*	LysSS	[46]
		*K. pneumoniae*	Dp42	[52]
Rabbits	Cholera-like diarrhea	*V. cholerae*	Phage cocktail	[63]
Dogs	Otitis externa	*P. aeruginosa*	Phage cocktail	[64]
	Urinary tract infections	*P. aeruginosa*	*Pbunavirus* PB1-like phages cocktail	[65]
		*E. coli*	5 promising single phages	[66]
	Wound infection	*P. aeruginosa*	*Pbunavirus* PB1-like phages cocktail	[65]
	Opportunistic bacteria in immunocompromised patients	*E. coli*	Phage HP3	[32]
Cats	Urinary tract infection	*E. coli*	5 promising single phages	[66]
Horses	Keratitis	*P. aeruginosa*	Phage cocktail	[67]
Bearded dragons	Pet-associated salmonellosis	*Salmonella* sp.	Felix O1 phage	[68]

## References

[1] Czaplewski L., Bax R., Clokie M., Dawson M., Fairhead H., Fischetti V.A., Foster S., Gilmore B.F., Hancock R.E.W., Harper D. (2016). Alternatives to Antibiotics—A Pipeline Portfolio Review. Lancet Infect. Dis..

[2] Machowska A., Stålsby Lundborg C. (2019). Drivers of Irrational Use of Antibiotics in Europe. Int. J. Environ. Res. Public Health.

[3] The World Medicines Situation 2011-Rational Use of Medicines. http://digicollection.org/hss/en/m/abstract/Js18064en/.

[4] Innes G.K., Randad P.R., Korinek A., Davis M.F., Price L.B., So A.D., Heaney C.D. (2020). External Societal Costs of Antimicrobial Resistance in Humans Attributable to Antimicrobial Use in Livestock. Ann. Rev. Public Health.

[5] WHO|Global Action Plan on Antimicrobial Resistance. http://www.who.int/antimicrobial-resistance/publications/global-action-plan/en/.

[6] Bandyopadhyay S., Samanta I. (2020). Antimicrobial Resistance in Agri-Food Chain and Companion Animals as a Re-Emerging Menace in Post-COVID Epoch: Low-and Middle-Income Countries Perspective and Mitigation Strategies. Front. Vet. Sci..

[7] Ginsburg A.S., Klugman K.P. (2020). COVID-19 Pneumonia and the Appropriate Use of Antibiotics. Lancet Glob. Health.

[8] Beović B., Doušak M., Ferreira-Coimbra J., Nadrah K., Rubulotta F., Belliato M., Berger-Estilita J., Ayoade F., Rello J., Erdem H. (2020). Antibiotic Use in Patients with COVID-19: A ‘Snapshot’ Infectious Diseases International Research Initiative (ID-IRI) Survey. J. Antimicrob. Chemother..

[9] Cully M. (2014). Public Health: The Politics of Antibiotics. Nature.

[10] Cuong N.V., Padungtod P., Thwaites G., Carrique-Mas J.J. (2018). Antimicrobial Usage in Animal Production: A Review of the Literature with a Focus on Low- and Middle-Income Countries. Antibiotics.

[11] Van Boeckel T.P., Pires J., Silvester R., Zhao C., Song J., Criscuolo N.G., Gilbert M., Bonhoeffer S., Laxminarayan R. (2019). Global Trends in Antimicrobial Resistance in Animals in Low- and Middle-Income Countries. Science.

[12] Ma Z., Lee S., Jeong K.C. (2019). Mitigating Antibiotic Resistance at the Livestock-Environment Interface: A Review. J. Microbiol. Biotechnol..

[13] World Health Organization, Department of Food Safety and Zoonoses (2017). World Health Organization WHO Guidelines on Use of Medically Important Antimicrobials in Food-Producing Animals.

[14] Angelakis E. (2017). Weight Gain by Gut Microbiota Manipulation in Productive Animals. Microb. Pathog..

[15] Guardabassi L., Schwarz S., Lloyd D.H. (2004). Pet Animals as Reservoirs of Antimicrobial-Resistant Bacteria. J. Antimicrob. Chemother..

[16] Pyzik E., Urban-Chmiel R., Radzki R.P. (2020). Experimental Phage Therapies in Companion Animals with Historical Review. Curr. Clin. Pharmacol..

[17] Lloyd D.H. (2007). Reservoirs of Antimicrobial Resistance in Pet Animals. Clin. Infect. Dis..

[18] Viertel T.M., Ritter K., Horz H.-P. (2014). Viruses versus Bacteria-Novel Approaches to Phage Therapy as a Tool against Multidrug-Resistant Pathogens. J. Antimicrob. Chemother..

[19] Weinbauer M.G. (2004). Ecology of Prokaryotic Viruses. FEMS Microbiol. Rev..

[20] Clark J.R., March J.B. (2006). Bacteriophages and Biotechnology: Vaccines, Gene Therapy and Antibacterials. Trends Biotechnol..

[21] Domingo-Calap P., Delgado-Martínez J. (2018). Bacteriophages: Protagonists of a Post-Antibiotic Era. Antibiotics.

[22] Guzmán M. (2015). The bacteriophage, one hundred years of significant findings. Biomédica.

[23] Furfaro L.L., Chang B.J., Payne M.S. (2017). Applications for Bacteriophage Therapy during Pregnancy and the Perinatal Period. Front. Microbiol..

[24] Haddad Kashani H., Schmelcher M., Sabzalipoor H., Seyed Hosseini E., Moniri R. (2017). Recombinant Endolysins as Potential Therapeutics against Antibiotic-Resistant Staphylococcus Aureus: Current Status of Research and Novel Delivery Strategies. Clin. Microbiol. Rev..

[25] Abedon S.T., Kuhl S.J., Blasdel B.G., Kutter E.M. (2011). Phage Treatment of Human Infections. Bacteriophage.

[26] Squires R. (2018). Bacteriophage Therapy for Management of Bacterial Infections in Veterinary Practice: What Was Once Old Is New Again. N. Z. Vet. J..

[27] Allen R.C., Pfrunder-Cardozo K.R., Meinel D., Egli A., Hall A.R. (2017). Associations among Antibiotic and Phage Resistance Phenotypes in Natural and Clinical Escherichia Coli Isolates. mBio.

[28] Chen L.-K., Kuo S.-C., Chang K.-C., Cheng C.-C., Yu P.-Y., Chang C.-H., Chen T.-Y., Tseng C.-C. (2017). Clinical Antibiotic-Resistant Acinetobacter Baumannii Strains with Higher Susceptibility to Environmental Phages than Antibiotic-Sensitive Strains. Sci. Rep..

[29] Payne R.J., Phil D., Jansen V.A. (2000). Phage Therapy: The Peculiar Kinetics of Self-Replicating Pharmaceuticals. Clin. Pharmacol. Ther..

[30] Miller-Ensminger T., Garretto A., Brenner J., Thomas-White K., Zambom A., Wolfe A.J., Putonti C. (2018). Bacteriophages of the Urinary Microbiome. J. Bacteriol..

[31] Melo L.D.R., Veiga P., Cerca N., Kropinski A.M., Almeida C., Azeredo J., Sillankorva S. (2016). Development of a Phage Cocktail to Control Proteus Mirabilis Catheter-Associated Urinary Tract Infections. Front. Microbiol..

[32] Chan B.K., Abedon S.T., Loc-Carrillo C. (2013). Phage Cocktails and the Future of Phage Therapy. Futur. Microbiol..

[33] Green S.I., Kaelber J.T., Ma L., Trautner B.W., Ramig R.F., Maresso A.W. (2017). Bacteriophages from ExPEC Reservoirs Kill Pandemic Multidrug-Resistant Strains of Clonal Group ST131 in Animal Models of Bacteremia. Sci. Rep..

[34] Fischer S., Kittler S., Klein G., Glünder G. (2013). Impact of a Single Phage and a Phage Cocktail Application in Broilers on Reduction of Campylobacter Jejuni and Development of Resistance. PLoS ONE.

[35] Yu L., Wang S., Guo Z., Liu H., Sun D., Yan G., Hu D., Du C., Feng X., Han W. (2018). A Guard-Killer Phage Cocktail Effectively Lyses the Host and Inhibits the Development of Phage-Resistant Strains of Escherichia Coli. Appl. Microbiol. Biotechnol..

[36] Biological Challenges of Phage Therapy and Proposed Solutions: A Literature Review. https://www.ncbi.nlm.nih.gov/pmc/articles/PMC6919273/.

[37] Malik D.J., Sokolov I.J., Vinner G.K., Mancuso F., Cinquerrui S., Vladisavljevic G.T., Clokie M.R.J., Garton N.J., Stapley A.G.F., Kirpichnikova A. (2017). Formulation, Stabilisation and Encapsulation of Bacteriophage for Phage Therapy. Adv. Colloid Interf. Sci..

[38] Sechter I., Touitou E., Donbrow M. (1989). The Influence of a Non-Ionic Surfactant on Rectal Absorption of Virus Particles. Arch. Virol..

[39] Bochkareva S.S., Karaulov A.V., Aleshkin A.V., Novikova L.I., Kiseleva I.A., Rubal’skii E.O., Mekhtiev E.R., Styshnev A.O., Zul’karneev E.R., Anurova M.N. (2020). Analysis of the Pharmacokinetics of Suppository Forms of Bacteriophages. Bull Exp. Biol. Med..

[40] Bodier-Montagutelli E., Morello E., L’Hostis G., Guillon A., Dalloneau E., Respaud R., Pallaoro N., Blois H., Vecellio L., Gabard J. (2017). Inhaled Phage Therapy: A Promising and Challenging Approach to Treat Bacterial Respiratory Infections. Exp. Opin. Drug Deliv..

[41] Chadha P., Katare O.P., Chhibber S. (2016). In Vivo Efficacy of Single Phage versus Phage Cocktail in Resolving Burn Wound Infection in BALB/c Mice. Microb. Pathog..

[42] Maciejewska B., Olszak T., Drulis-Kawa Z. (2018). Applications of Bacteriophages versus Phage Enzymes to Combat and Cure Bacterial Infections: An Ambitious and Also a Realistic Application?. Appl. Microbiol. Biotechnol..

[43] São-José C. (2018). Engineering of Phage-Derived Lytic Enzymes: Improving Their Potential as Antimicrobials. Antibiotics (Basel).

[44] Ferriol-González C., Domingo-Calap P. (2020). Phages for Biofilm Removal. Antibiotics.

[45] Carvalho C., Costa A.R., Silva F., Oliveira A. (2017). Bacteriophages and Their Derivatives for the Treatment and Control of Food-Producing Animal Infections. Crit. Rev. Microbiol..

[46] Kim S., Lee D.-W., Jin J.-S., Kim J. (2020). Antimicrobial Activity of LysSS, a Novel Phage Endolysin, against Acinetobacter Baumannii and Pseudomonas Aeruginosa. J. Glob. Antimicrob. Resist..

[47] Drulis-Kawa Z., Majkowska-Skrobek G., Maciejewska B. (2015). Bacteriophages and Phage-Derived Proteins--Application Approaches. Curr. Med. Chem..

[48] Wang I.N., Smith D.L., Young R. (2000). Holins: The Protein Clocks of Bacteriophage Infections. Annu. Rev. Microbiol..

[49] Criscuolo E., Spadini S., Lamanna J., Ferro M., Burioni R. (2017). Bacteriophages and Their Immunological Applications against Infectious Threats. J. Immunol. Res..

[50] Pires D.P., Oliveira H., Melo L.D.R., Sillankorva S., Azeredo J. (2016). Bacteriophage-Encoded Depolymerases: Their Diversity and Biotechnological Applications. Appl. Microbiol. Biotechnol..

[51] Pires D., Melo L., Vilas Boas D., Sillankorva S., Azeredo J. (2017). Phage Therapy as an Alternative or Complementary Strategy to Prevent and Control Biofilm-Related Infections. Curr. Opin. Microbiol..

[52] Wang C., Li P., Niu W., Yuan X., Liu H., Huang Y., An X., Fan H., Zhangxiang L., Mi L. (2019). Protective and Therapeutic Application of the Depolymerase Derived from a Novel KN1 Genotype of Klebsiella Pneumoniae Bacteriophage in Mice. Res. Microbiol..

[53] Ngassam-Tchamba C., Duprez J.N., Fergestad M., De Visscher A., L’Abee-Lund T., De Vliegher S., Wasteson Y., Touzain F., Blanchard Y., Lavigne R. (2020). In Vitro and in Vivo Assessment of Phage Therapy against Staphylococcus Aureus Causing Bovine Mastitis. J. Glob. Antimicrob. Resist..

[54] Breyne K., Honaker R.W., Hobbs Z., Richter M., Żaczek M., Spangler T., Steenbrugge J., Lu R., Kinkhabwala A., Marchon B. (2017). Efficacy and Safety of a Bovine-Associated Staphylococcus Aureus Phage Cocktail in a Murine Model of Mastitis. Front. Microbiol..

[55] Titze I., Krömker V. (2020). Antimicrobial Activity of a Phage Mixture and a Lactic Acid Bacterium against Staphylococcusaureus from Bovine Mastitis. Vet. Sci..

[56] Santos T.M.A., Gilbert R.O., Caixeta L.S., Machado V.S., Teixeira L.M., Bicalho R.C. (2010). Susceptibility of Escherichia Coli Isolated from Uteri of Postpartum Dairy Cows to Antibiotic and Environmental Bacteriophages. Part II: In Vitro Antimicrobial Activity Evaluation of a Bacteriophage Cocktail and Several Antibiotics. J. Dairy Sci..

[57] Qureshi S., Saxena H.M., Imam N., Kashoo Z., Sharief Banday M., Alam A., Malik M.Z., Ishrat R., Bhat B. (2018). Isolation and Genome Analysis of a Lytic Pasteurella Multocida Bacteriophage PMP-GAD-IND. Lett. Appl. Microbiol..

[58] Wall S.K., Zhang J., Rostagno M.H., Ebner P.D. (2010). Phage Therapy to Reduce Preprocessing Salmonella Infections in Market-Weight Swine. Appl. Environ. Microbiol..

[59] Milho C., Silva M.D., Sillankorva S., Harper D.R., Harper D.R., Abedon S.T., Burrowes B.H., McConville M.L. (2019). Biofilm Applications of Bacteriophages. Bacteriophages.

[60] Waters E.M., Neill D.R., Kaman B., Sahota J.S., Clokie M.R.J., Winstanley C., Kadioglu A. (2017). Phage Therapy Is Highly Effective against Chronic Lung Infections with Pseudomonas Aeruginosa. Thorax.

[61] Wang J.-L., Kuo C.-F., Yeh C.-M., Chen J.-R., Cheng M.-F., Hung C.-H. (2018). Efficacy of Φkm18p Phage Therapy in a Murine Model of Extensively Drug-Resistant Acinetobacter Baumannii Infection. Infect. Drug Resist..

[62] Takemura-Uchiyama I., Uchiyama J., Osanai M., Morimoto N., Asagiri T., Ujihara T., Daibata M., Sugiura T., Matsuzaki S. (2014). Experimental Phage Therapy against Lethal Lung-Derived Septicemia Caused by Staphylococcus Aureus in Mice. Microb. Infect..

[63] Yen M., Cairns L.S., Camilli A. (2017). A Cocktail of Three Virulent Bacteriophages Prevents Vibrio Cholerae Infection in Animal Models. Nat. Commun..

[64] Hawkins C., Harper D., Burch D., Anggård E., Soothill J. (2010). Topical Treatment of Pseudomonas Aeruginosa Otitis of Dogs with a Bacteriophage Mixture: A before/after Clinical Trial. Vet. Microbiol..

[65] Fujiki J., Furusawa T., Munby M., Kawaguchi C., Matsuda Y., Shiokura Y., Nakamura K., Nakamura T., Sasaki M., Usui M. (2020). Susceptibility of Pseudomonas Aeruginosa Veterinary Isolates to Pbunavirus PB1-like Phages. Microbiol. Immunol..

[66] Freitag T., Squires R.A., Schmid J. (2008). Naturally Occurring Bacteriophages Lyse a Large Proportion of Canine and Feline Uropathogenic Escherichia Coli Isolates in Vitro. Res. Veter. Sci..

[67] Furusawa T., Iwano H., Hiyashimizu Y., Matsubara K., Higuchi H., Nagahata H., Niwa H., Katayama Y., Kinoshita Y., Hagiwara K. (2016). Phage Therapy Is Effective in a Mouse Model of Bacterial Equine Keratitis. Appl. Environ. Microbiol..

[68] Renfert K., Rabsch W., Fruth A., Speck S., Pees M. (2019). The Use of a Salmonella Bacteriophage in Bearded Dragons: Application, Passage Time and Reisolation. Tierarztl. Prax. Ausg. K Kleintiere Heimtiere.

[69] Dec M., Wernicki A., Urban-Chmiel R. (2020). Efficacy of Experimental Phage Therapies in Livestock. Anim. Health Res. Rev..

[70] McCarthy A.J., Lindsay J.A. (2010). Genetic Variation in Staphylococcus Aureus Surface and Immune Evasion Genes Is Lineage Associated: Implications for Vaccine Design and Host-Pathogen Interactions. BMC Microbiol..

[71] Arias C.A., Murray B.E. (2009). Antibiotic-Resistant Bugs in the 21st Century—A Clinical Super-Challenge. N. Engl. J. Med..

[72] Halasa T., Huijps K., Østerås O., Hogeveen H. (2007). Economic Effects of Bovine Mastitis and Mastitis Management: A Review. Vet. Quart..

[73] Geng H., Zou W., Zhang M., Xu L., Liu F., Li X., Wang L., Xu Y. (2020). Evaluation of Phage Therapy in the Treatment of Staphylococcus Aureus-Induced Mastitis in Mice. Folia Microbiol. (Praha).

[74] Sheldon I.M., Cronin J., Goetze L., Donofrio G., Schuberth H.-J. (2009). Defining Postpartum Uterine Disease and the Mechanisms of Infection and Immunity in the Female Reproductive Tract in Cattle. Biol. Reprod..

[75] Piccardi M., Romero G., Veneranda G., Castello E., Romero D., Balzarini M., Bó G.A. (2016). Effect of Puerperal Metritis on Reproductive and Productive Performance in Dairy Cows in Argentina. Theriogenology.

[76] Zduńczyk S., Janowski T. (2020). Bacteriophages and Associated Endolysins in Therapy and Prevention of Mastitis and Metritis in Cows: Current Knowledge. Anim. Reprod. Sci..

[77] Machado V.S., Bicalho M.L.S., Pereira R.V., Caixeta L.S., Bittar J.H.J., Oikonomou G., Gilbert R.O., Bicalho R.C. (2012). The Effect of Intrauterine Administration of Mannose or Bacteriophage on Uterine Health and Fertility of Dairy Cows with Special Focus on Escherichia Coli and Arcanobacterium Pyogenes. J. Dairy Sci..

[78] Meira E.B.S., Rossi R.S., Teixeira A.G., Kaçar C., Oikonomou G., Gregory L., Bicalho R.C. (2013). The Effect of Prepartum Intravaginal Bacteriophage Administration on the Incidence of Retained Placenta and Metritis. J. Dairy Sci..

[79] Fairbrother J.M., Nadeau E., Gyles C.L. (2005). Escherichia Coli in Postweaning Diarrhea in Pigs: An Update on Bacterial Types, Pathogenesis, and Prevention Strategies. Anim. Health Res. Rev..

[80] Wernicki A., Nowaczek A., Urban-Chmiel R. (2017). Bacteriophage Therapy to Combat Bacterial Infections in Poultry. Virol. J..

[81] Hoai T.D., Nishiki I., Fujiwara A., Yoshida T., Nakai T. (2019). Comparative Genomic Analysis of Three Lytic Lactococcus Garvieae Phages, Novel Phages with Genome Architecture Linking the 936 Phage Species of Lactococcus Lactis. Mar. Genom..

[82] Intralytix, Inc. http://www.intralytix.com/index.php?page=prod&id=1.

[83] PhageGuard-The Natural Solution for Food Safety. http://phageguard.com/.

[84] Rhys-Davies L., Ogden J. (2020). Vets’ and Pet Owners’ Views About Antibiotics for Companion Animals and the Use of Phages as an Alternative. Front. Vet. Sci..

[85] Lynch S.A., Helbig K.J. (2021). The Complex Diseases of Staphylococcus Pseudintermedius in Canines: Where to Next?. Vet. Sci..

[86] McCallin S., Sacher J.C., Zheng J., Chan B.K. (2019). Current State of Compassionate Phage Therapy. Viruses.

[87] Fauconnier A. (2019). Phage Therapy Regulation: From Night to Dawn. Viruses.

[88] Russian Pharmacopoeia OFS.1.7.1.0002.15 Bacteriophages are Therapeutic and Prophylactic. http://pharmacopoeia.ru/ofs-1-7-1-0002-15-bakteriofagi-lechebno-profilakticheskie/.

[89] Parfitt T. (2005). Georgia: An Unlikely Stronghold for Bacteriophage Therapy. Lancet.

[90] Fauconnier A. (2017). Regulating Phage Therapy: The Biological Master File Concept Could Help to Overcome Regulatory Challenge of Personalized Medicines. EMBO Rep..

[91] Actualité-Phagothérapie: L’ANSM Annonce la Création d’un Comité Scientifique Spécialisé Temporaire (CSST) Intitulé “Phagothérapie–Retour d’Expérience et Perspectives”-ANSM. https://ansm.sante.fr/actualites/phagotherapie-lansm-annonce-la-creation-dun-comite-scientifique-specialise-temporaire-csst-intitule-phagotherapie-retour-dexperience-et-perspectives.

[92] Alsaadi A., Beamud B., Easwaran M., Abdelrahman F., El-Shibiny A., Alghoribi M.F., Domingo-Calap P. (2021). Learning From Mistakes: The Role of Phages in Pandemics. Front. Microbiol..

[93] Adaptive Phage Therapeutics, Inc. Expanded Access Study of Phage Treatment in Covid-19 Patients on Anti-Microbials for Pneumonia or Bacteremia/Septicemia Due to *A. baumannii*, *P. aeruginosa* or *S. aureus*. http://clinicaltrials.gov/ct2/show/NCT04636554.

[94] Gigante A., Atterbury R.J. (2019). Veterinary Use of Bacteriophage Therapy in Intensively-Reared Livestock. Virol. J..

[95] Answer to Question No E-002838/18. https://www.europarl.europa.eu/doceo/document/E-8-2018-002838-ASW_EN.html.

